# Ionic complexation improves wound healing in deep second-degree burns and reduces in-vitro ciprofloxacin cytotoxicity in fibroblasts

**DOI:** 10.1038/s41598-022-19969-w

**Published:** 2022-09-26

**Authors:** María Florencia Sanchez, María Laura Guzman, Jesica Flores-Martín, Mariano Cruz Del Puerto, Carlos Laino, Elio Andrés Soria, Ana Carolina Donadio, Susana Genti-Raimondi, María Eugenia Olivera

**Affiliations:** 1 Unidad de Investigación y Desarrollo en Tecnología Farmacéutica (UNITEFA), CONICET and Departamento de Ciencias Farmacéuticas, Facultad de Ciencias Químicas, Universidad Nacional de Córdoba, Ciudad Universitaria, 5000 Córdoba, Argentina; 2 Departamento de Bioquímica Clínica and Consejo Nacional de Investigaciones Científicas y Tecnológicas (CONICET), Centro de Investigaciones en Bioquímica Clínica e Inmunología (CIBICI), Universidad Nacional de Córdoba, Facultad de Ciencias Químicas, Ciudad Universitaria, X5000HUA Córdoba, Argentina; 3 Instituto de Biotecnología, Centro de Investigación e Innovación Tecnológica (CENIIT), Universidad Nacional de La Rioja, 5300 La Rioja, Argentina; 4 Instituto de Investigaciones en Ciencias de la Salud (INICSA), CONICET and Facultad de Ciencias Médicas, Universidad Nacional de Córdoba, Ciudad Universitaria, 5000 Córdoba, Argentina

**Keywords:** Skin diseases, Pharmacodynamics, Drug delivery, Molecular biology

## Abstract

The development of new treatments capable of controlling infections and pain related to burns continues to be a challenge. Antimicrobials are necessary tools, but these can be cytotoxic for regenerating cells. In this study, antibiotic-anesthetic (AA) smart systems obtained by ionic complexation of polyelectrolytes with ciprofloxacin and lidocaine were obtained as films and hydrogels. Ionic complexation with sodium alginate and hyaluronate decreased cytotoxicity of ciprofloxacin above 70% in a primary culture of isolated fibroblasts (p < 0.05). In addition, the relative levels of the proteins involved in cell migration, integrin β1 and p-FAK, increased above 1.5 times (p < 0.05) with no significant differences in cell mobility. Evaluation of the systems in a deep second-degree burn model revealed that reepithelization rate was AA-films = AA-hydrogels > control films > no treated > reference cream (silver sulfadiazine cream). In addition, appendage conservation and complete dermis organization were achieved in AA-films and AA-hydrogels. Encouragingly, both the films and the hydrogels showed a significantly superior performance compared to the reference treatment. This work highlights the great potential of this smart system as an attractive dressing for burns, which surpasses currently available treatments.

## Introduction

Burns are a global public health problem, especially in low- and middle-income countries, where approximately 180,000 deaths are accounted annually. Also, non-fatal burns cause significant morbidity as prolonged hospitalization, disfigurement and disability^1,2^. Wounds are susceptible to infections, and their treatment is important because infections can slow down the rate of wound healing. Concerning burns, most non-complicated superficial second degree ones will heal spontaneously or after a conservative treatment^3^. In deep partial thickness burns, there is damage to the deeper structures of the dermis, involving sweat glands and hair follicles, and these take more than 2–3 weeks to heal. Thus, they are more likely to result in scarring with functional and psychological consequences^4^. Therefore, a topical therapy that supports sustained antimicrobial activity would prevent infections and facilitate wound healing^5^. The antimicrobial choice should be taken with caution, since, in addition to its bactericidal activity, antimicrobials can produce eukaryotic skin cell death, thereby preventing tissue regeneration. The level of toxicity depends on its specificity and its mechanism of action^6^.

The gold standard for topical treatment of second-degree burns is silver sulfadiazine. However, its use has been systematically questioned, mainly due to the lack of selectivity of its cytotoxic mechanisms^7,8^. In this sense, new nanosilver-based dressings are claimed as more biocompatible and convenient since they do not require a daily change, but are still cytotoxic to skin cells and deficient in promoting wound healing^6,7,9,10^. It has also been described that silver is beneficial in infected wounds only for the first few days/weeks, after which non-silver dressings should be used^11^. Chitosan-capped silver nanoparticles showed a better potential for healing burn wounds than the negative control and silver sulfadiazine groups^12^. However, other authors found no difference between nanosilver-based dressings and 1% silver sulfadiazine regarding efficacy and safety outcomes in patients with partial thickness thermal burns^13^. Moreover, the efficacy and safety of the combination of nano-silver dressings with recombinant human epidermal growth factor are still inconclusive^14^. Therefore, there is still room for improvements in burn dressing formulations.

Biomaterials, such as polyelectrolytes (PE), can be utilized as carriers to obtain systems with a modified release of antimicrobials. This specific and regulated release strategy can reduce the cytotoxicity of the antimicrobials^15^. In this sense, ciprofloxacin (Cip)-based PE dressings were proposed for wound healing and have shown good biocompatibility and antimicrobial properties^16,17^. In previous studies, an antibiotic-anaesthetic hydrogel (AA-hydrogel) and antibiotic-anaesthetic film (AA-films) were developed. In both cases, the ionic interactions established between the PE and drugs allowed sustained-release systems to be obtained with potential applications in wound healing. AA-hydrogel revealed faster reepithelization of superficial second degree burns than those treated with silver sulfadiazine, as well as providing pain relief^18^. Although the obtaining of AA-films requires a more complex procedure than that of the hydrogels (with a deaeration and drying steps), they have the advantage of easy and non-invasive application and dose accuracy^19^. In addition, AA-films are transparent, compatible with wound skin pH, highly water vapor permeable, and can absorb twice their weight in fluid while maintaining its integrity. The films also act as a physical barrier for microorganisms, and the in vitro antimicrobial efficacy against *S. aureus* and *P. aeruginosa* has been demonstrated^20^. However, neither of these two systems has been evaluated in deep second-degree burns. Moreover, the impact of PE-drug complexation on eukaryotic cells is unknown.

The objective of this work was to evaluate the wound healing performance of these AA-systems and compare them with the reference treatment, using an experimental model of deep second-degree burns. In addition, we studied the viability and cell migration in a primary culture of human dermal fibroblasts. We found that AA-hydrogels and films facilitated wound repair with safety and efficacy, which means they could be considered potential treatments in the clinic.

## Results and discussion

### Cytotoxicity of Cip, PE and PE-Cip in HDF cells

#### PE cell compatibility

The cell compatibility of wound dressings is usually evaluated in the final system to be compared with that of the cargo. However, the inherent cytotoxicity of each single component can help to understand its protective potential. With that in mind, we evaluated HDF cell viability when these cells were exposed to treatments with low (0.01% p/p, PE_L_) or high (0.1% p/p, PE_H_) concentrations of carbomer (CB), sodium alginate (SA), and sodium hyaluronate (SH).

**Figure 1:**
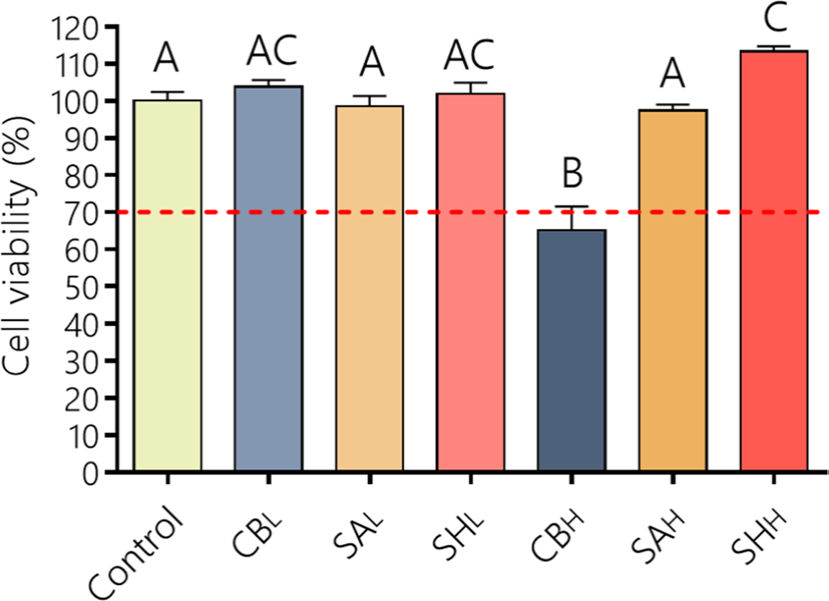
Effect of PE_L_ and PE_H_ on CV% of HDF cells after 24 h of treatment. The CV% was calculated from Eq. (1). Values are expressed as the means ± their standard errors (n = 8). Differences between the treatments were evaluated with ANOVA and Tukey's post-test (p < 0.05), with different capital letters above the bars indicating significant differences. The dotted line determines the cell viability limit allowed by ISO 10993 5 (CV ≥ 70%). Plotted with GraphPad Prism v.7.00 software (GraphPad, USA, https://www.graphpad.com/).

The PE_L_ concentrations did not modify the cell viability related to the control (Fig. 1). In the case of SH_H_, the HDF cell viability was even slightly increased (15%, p < 0.05), suggesting excellent biocompatibility properties. In agreement, Guo et al.^21^ observed the same tendency in the mouse fibroblast line L929. The increase in HDF cell viability in the presence of SH may be related to its endogenous nature, since it is the most relevant structural element of the extracellular matrix^22^. In fact, HDFs have SH primary cell receptors (CD44 and RHAMM) that are involved in the modulation of complex biological functions such as cell migration and proliferation, inflammation and tumorigenicity^23^.

Good cell compatibility was also evident in the presence of SA_H_, which produced no changes in HDF cell viability. These results are not in agreement with Guo et al.^21^, where SA 0.1% (SA_H_) reduced viability to approximately 70%. Such differences could be due to the cell line used (mouse fibroblast L929), to differences in viscosity or in monomeric composition (manuronic:guluronic ratio), or to combination of both.

In the case of CB_H_, cell viability decreased to 65%, which implied a low in vitro biocompatibility. Similar results were observed by Guo et al.^21^ in a mouse fibroblast line L929, which could be assigned to the characteristic higher viscosity of synthetic polyelectrolytes, which generates three-dimensional structures covering living cells and hindering the arrival of culture medium nutrients, thereby reducing the metabolic activity or blocking the transport of vital molecules to cells^24^. However, it is important to note that this in vitro phenomenon may not necessarily occur in an in vivo condition, where the nutrition of the cells in a tissue comes from the extracellular matrix components. In fact, the healing of second-degree superficial burns treated with an hydrogel of carbomer combined with Cip and lidocaine (CbNaCipLid) has shown a fast and complete regeneration^18^. In addition, a CB hydrogel showed to be nontoxic when was applied to rabbit burn wounds, improving tissue perfusion and reducing the area of necrotic tissue^25^. Moreover, it has been proposed as a convenient vehicle for recombinant human acidic fibroblast growth factor (rh-aFGF) for wound healing^26^.

#### Cell compatibility of Cip and PE-Cip complexes

**Figure 2:**
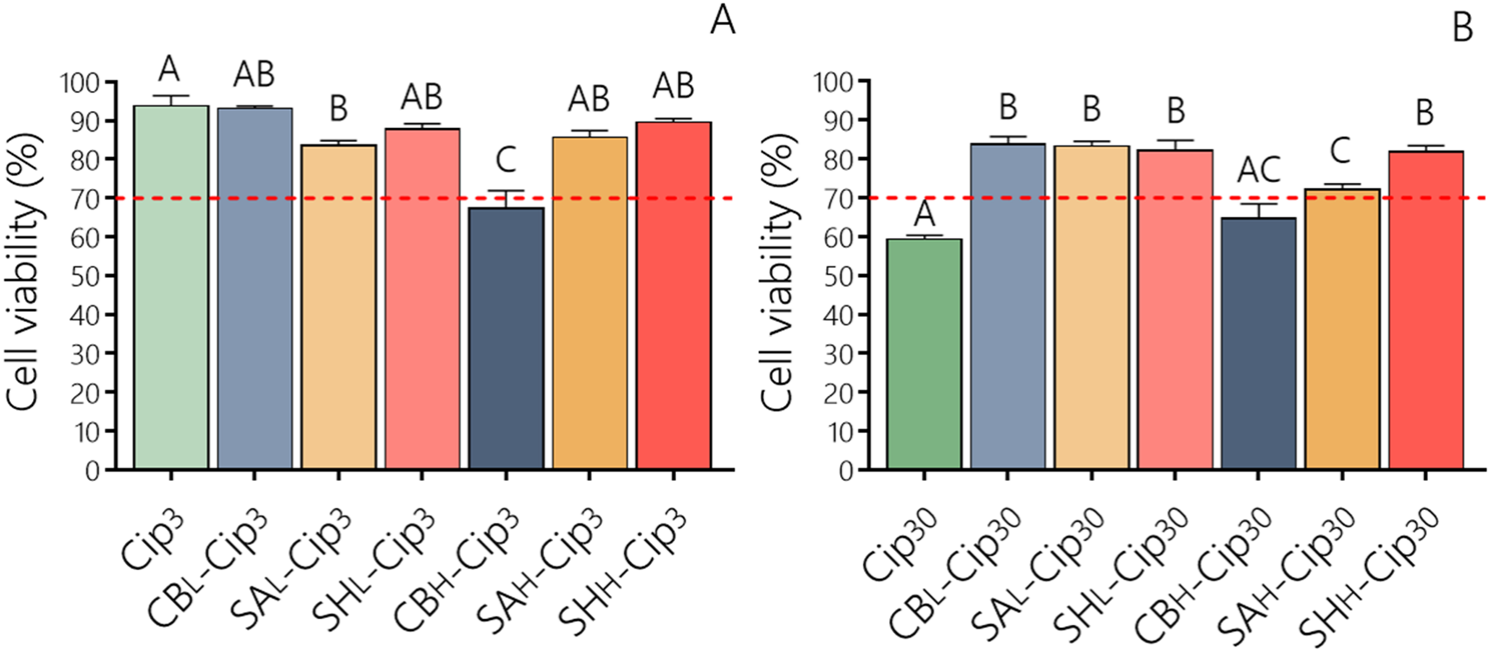
Cell viability of HDFs after 24 h of treatment with (**A**) Cip_3_ and its complexes with PE_L_ or PE_H,_ and (**B**) Cip_30_ and its complexes with PE_L_ or PE_H_. The CV% was calculated according to Eq. (1). Values are expressed as the means ± their standard errors (n = 8). Differences between the treatments were evaluated by ANOVA and Tukey's post-test (p < 0.05), with different capital letters above the bars indicating significant differences. The dotted line determines the cell viability limit allowed by ISO 10993 5 (CV ≥ 70%). Plotted with GraphPad Prism v.7.00 software (GraphPad, USA, https://www.graphpad.com/).

The addition of Cip_3_ to cell cultures produced a non-cytotoxic effect (Fig. 2A), whereas Cip_30_ treatment significantly reduced cell viability by up to 60% (Fig. 2B). In this regard, it has been reported that Cip inhibits cellular functions, thus preventing the growth of mammalian cell lines. Cip also induces mitochondrial dysfunction and oxidative damage in a dose-dependent manner. However, this toxic effect can be counteracted by the exogenous incorporation of vitamin E as an antioxidant^27,28^.

Next, we evaluated the HDF cell viability of Cip at 3 µg/mL or 30 µg/mL in the presence of PE. No cytotoxic effect was observed when Cip 3 µg/mL was combined with PE, except for CB_H_-Cip_3_, which followed the same pattern as CB_H_ alone. A slight cell viability reduction was observed for SA_L_-Cip_3_ and SA_H_-Cip_3_. However, these were not cytotoxic since the values were higher than 70% (Fig. 2A).

Interestingly, at higher cytotoxic concentrations of Cip_30_, a significant increase in HDF cell viability was revealed in the presence of PE_L_ and PE_H_, with the only exception being CB_H_-Cip_30_, whose non-significant increase in viability was still below 70% (Fig. 2B).

The increase in HDF cell viability by PE-Cip complexes with respect to Cip alone suggests that these PE have a protective effect, which could be due to the ability of SA and SH to counteract the cellular damage produced by the reactive oxygen species arising from the presence of Cip^29,30^. In addition, the electrostatic PE-Cip interaction can reduce free Cip concentrations in the medium^31^, so a combination of both mechanisms cannot be ruled out. Similar results were reported by Sauerová et al.^32^, which found that the surfactant-induced cytotoxicity of cationic surfactants on human osteoblasts, keratinocytes and fibroblasts was reduced by complexation with hyaluronic acid, with electrostatic attraction between the oppositely charged groups being the principal interaction force.

### Effect of PE and its PE-Cip complexes on HDF cell migration

**Figure 3:**
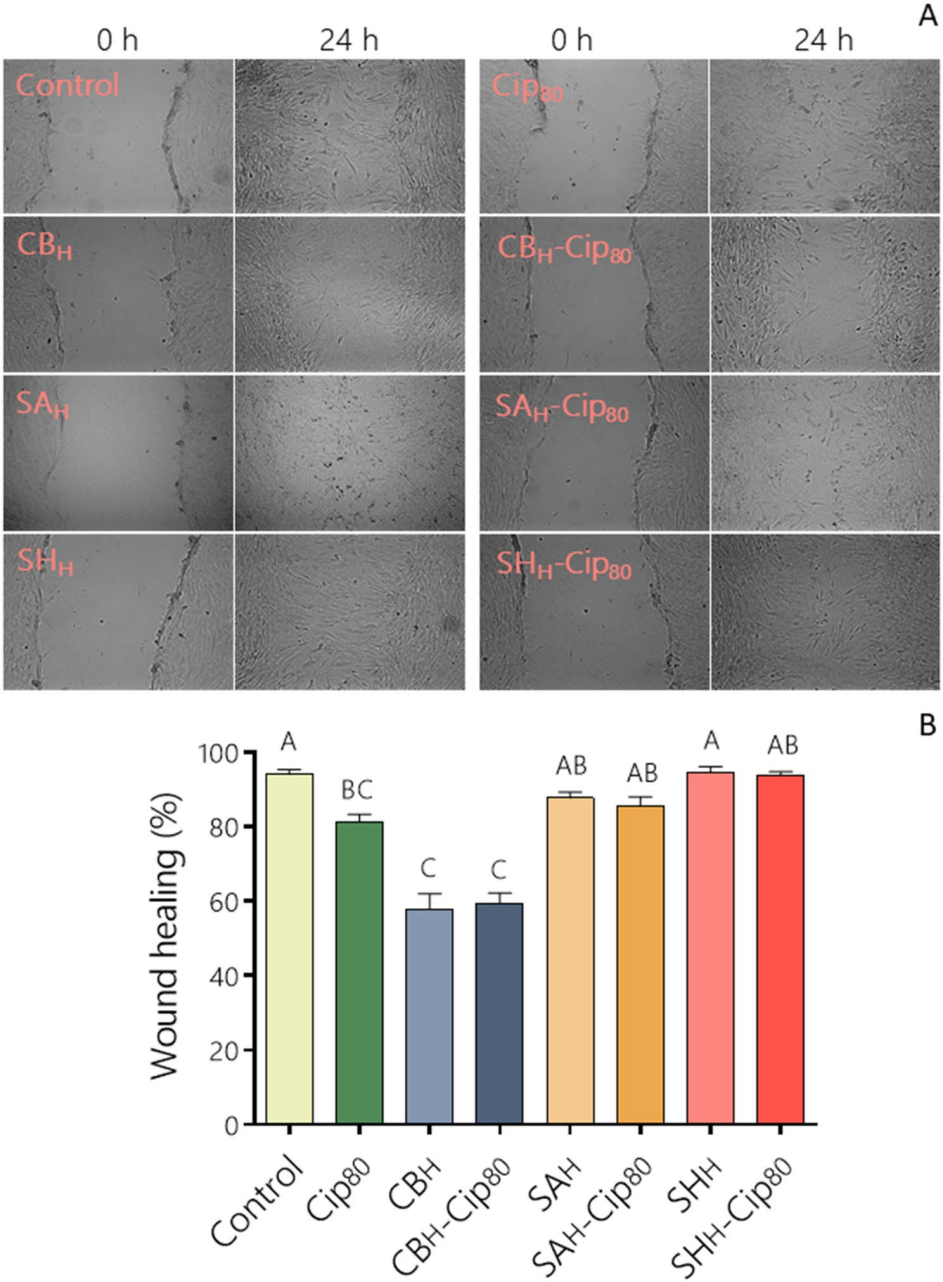
Effect of CB, SA, SH, or PE-Cip complexes on cell migration. (**A**) Representative images of wound healing assays performed on HDF cells treated as indicated. An open furrow was generated by scratching confluent cells using a pipette tip, and cells images were obtained at the initial time and after 24 h. (**B**) The distance between furrow edges in control or treated cells of three independent experiments was measured and presented graphically as the percentage of the initial distance (0 h) according Eq. (2); p < 0.05 compared to control cells. Different capital letters above the bars indicating significant differences. Plotted with GraphPad Prism v.7.00 software (GraphPad, USA, https://www.graphpad.com/).

To analyze the effect of PE alone and its Cip complexes on HDF cell migration, wound healing assays were performed. As shown in Fig. 3A, the cell treatment with SA or SH at high doses or in the presence of Cip_80_ did not alter the cell ability to heal the wound compared with the control; whereas, the addition of CB, Cip_80,_ or CB-Cip_80_ to the cell culture significantly decreased the cell migration compared to the control. The quantitative analyses of pooled data of three independent experiments are shown in Fig. 3B. Consistent with the Cip effect on HDF cells, Chen et al.^33^ reported that Cip also notably reduced the migration of human corneal fibroblasts. The decrease in the cell migration observed for CB alone or with Cip_80_ may have been due to a Cip cytotoxic effect on the cells (see Figs. 1 and 2), although an impact of the high viscosity of CB cannot be discarded. Related to this, it has been reported that cell mobility is directly reduced when cells are grown in a medium with high viscosity^34^.

### Effect of PE and its PE-Cip complexes on the protein expression implicated in cell migration

**Figure 4:**
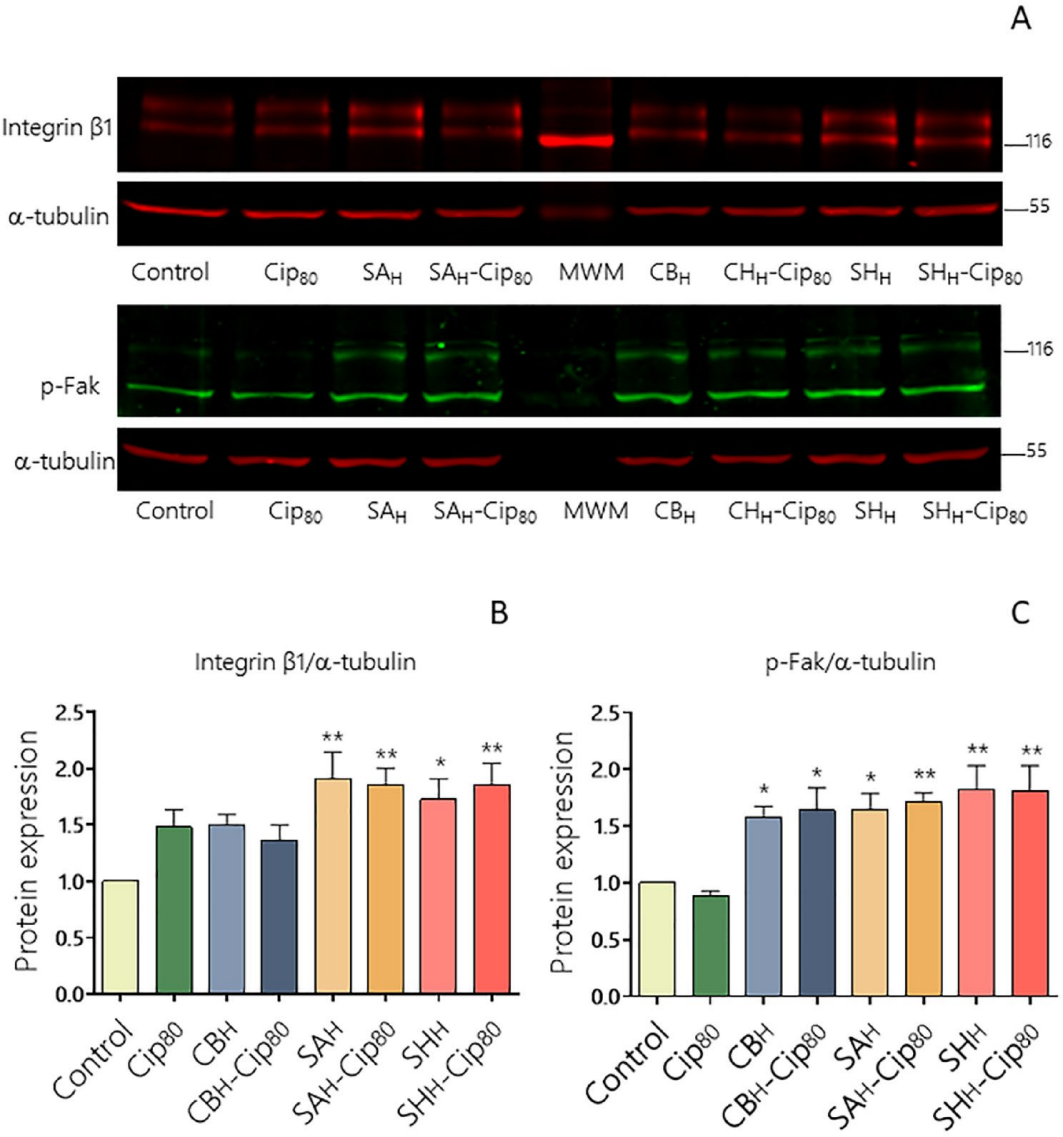
Effect of Cip, PE or PE-Cip complexes on integrin β1 and p-FAK protein expression. (**A**) Protein expression was analyzed by Western blot assays from cell lysates (100 µg/lane) of HDF-treated cells electrophoresed on a 7.5% SDS-PAGE and transferred to a nitrocellulose filter. Filters were incubated with anti-integrin β1, anti-p-FAK or anti-α-tubulin antibodies. Representative blot of three independent experiments with similar results is shown. Cropped blots from different parts of the same gel are separated by white spaces. (**B**, **C**) The bar graphs represent the densitometric quantification of protein levels in treated cells normalized to α-tubulin of three separate experiments compared to the corresponding normalized protein levels in control cells defined as 1 (mean ± SEM). Differences in treatments were evaluated using ANOVA with Dunnett's post-test *p < 0.05; **p < 0.01. Plotted with GraphPad Prism v.7.00 software (GraphPad, USA, https://www.graphpad.com/).

Integrin β1 and p-FAK is a signaling pathway involved in new focal adhesions formation and cell migration^35^. To establish the effect of the different treatments on the expression of the main proteins involved in cell migration, western blot assays were performed. Figure 4A and Supplementary Fig. S1 show representative images of integrin β1 and p-FAK expression in HDF cell cultures. The results indicate that cells exposed to Cip_80_, the CB_H_ dose or its CB_H_-Cip_80_ complex presented similar integrin β1 levels to the control cells; whereas cells treaded with SA_H_ and SH_H_ of their respective PE-Cip_80_ complexes had significantly increased integrin β1 levels compared to the control (Fig. 4B). Regarding p-FAK expression, all the treatments revealed increased levels, related to the Cip_80_ exposed cells (Fig. 4C).

These data are consistent with those observed with the in vitro wound healing assays. In addition, they suggest that these in vivo treatments may be accelerating wound closure. One of the first proteins that was identified downstream of integrin stimulation is FAK. This is a nonreceptor tyrosine kinase that localizes to the sites of integrin-mediated adhesion to the ECM. Activation of FAK results in increased cell motility and survival, as well as other cell responses involved in wound healing^36^. Taken together, these findings suggest that PE-Cip complexes are triggering a signal transduction cascade that promotes focal adhesions and regulates cell mobility. Although the in vitro systems cannot be directly extrapolated to the in vivo conditions, since it is not possible to reproduce exhaustively the tissue physiology, the in vitro test conditions provide greater sensitivity to antimicrobial toxicity than would be expected to found in in vivo systems.

### Wound-healing in vivo test

Although most non-complex burn injuries will heal spontaneously or after a conservative treatment^3^, several studies have shown that wounds that take more than 2–3 weeks to heal are more likely to result in scarring with functional and psychological consequences^4^. The management of burn wounds has a considerable influence on the time taken for the wound to heal^8^, so a good initial care will have a positive influence on the outcome^37^.

The healing process is divided into hemostasis, inflammation, proliferation, and remodeling phases. A faster closure involves cellular proliferation and migration, which is critical for restoring the barrier function and for minimizing the risk of wound infections^6, 8^. In overlapped with epithelization, dermal regeneration is mainly carried out by the migration and proliferation of fibroblasts and connective tissue. In addition, collagen and some other components of the extracellular matrix are also required for the wound to close efficiently.

There is an interplay between epidermis and dermis healing^38^, and a rapid dermal recovery will promote epidermal closure by providing its trophic support. The epithelial closure will prevent both the entry of microorganisms into dermis and the loss of extracellular water and solutes through the wound, which will allow a healthy dermal reorganization. Nonetheless, a fibrotic over-response can deleteriously lead to dense skin scars. The dermis also contributes to appendage regeneration and the biomechanical properties of the regenerated skin, which affect its quality^39^. If the new tissue is very different to the normal skin, the functionality will be compromised^37^, with poor aesthetic results and psychological sequels for patients^4^.

In this work, microscopic and macroscopic evaluation of the wound healing was performed. No signs of pain or general discomfort were observed in any animal during the trial, with the behavior (in terms of food and water intake and daily activity) being normal. In addition, no clinical signs of infection and inflammatory complications, such as, redness, heat and swelling were observed.

#### Microscopic wound healing study

**Figure 5:**
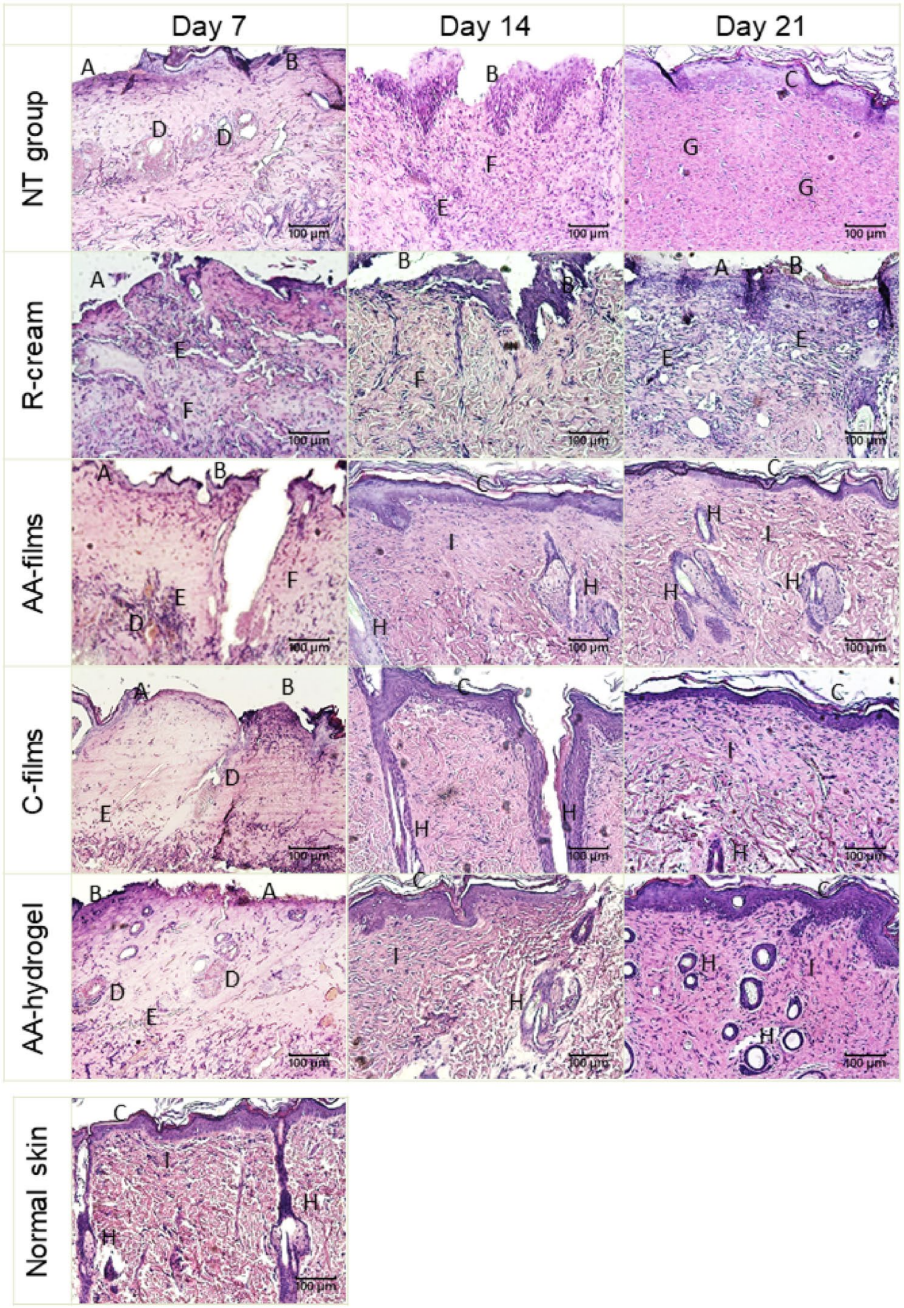
Representative photomicrographs of burn wounds for the NT group and after treatment with R-cream, AA-films, C-films or AA-hydrogel at days 7, 14, and 28 post wounding. H&E (× 100, scale bars: 100 μm). (**A**) Absent epidermis, (**B**) epidermis in regeneration, (**C**) continuous epidermis, (**D**) hemorrhagic appendages, (**E**) inflammatory cells, (**F**) dermal disorganization, (**G**) fibrous dermis, (**H**) appendages (hair follicles or glands), (**I**) differentiated papillary and reticular dermis.

**Figure 6:**
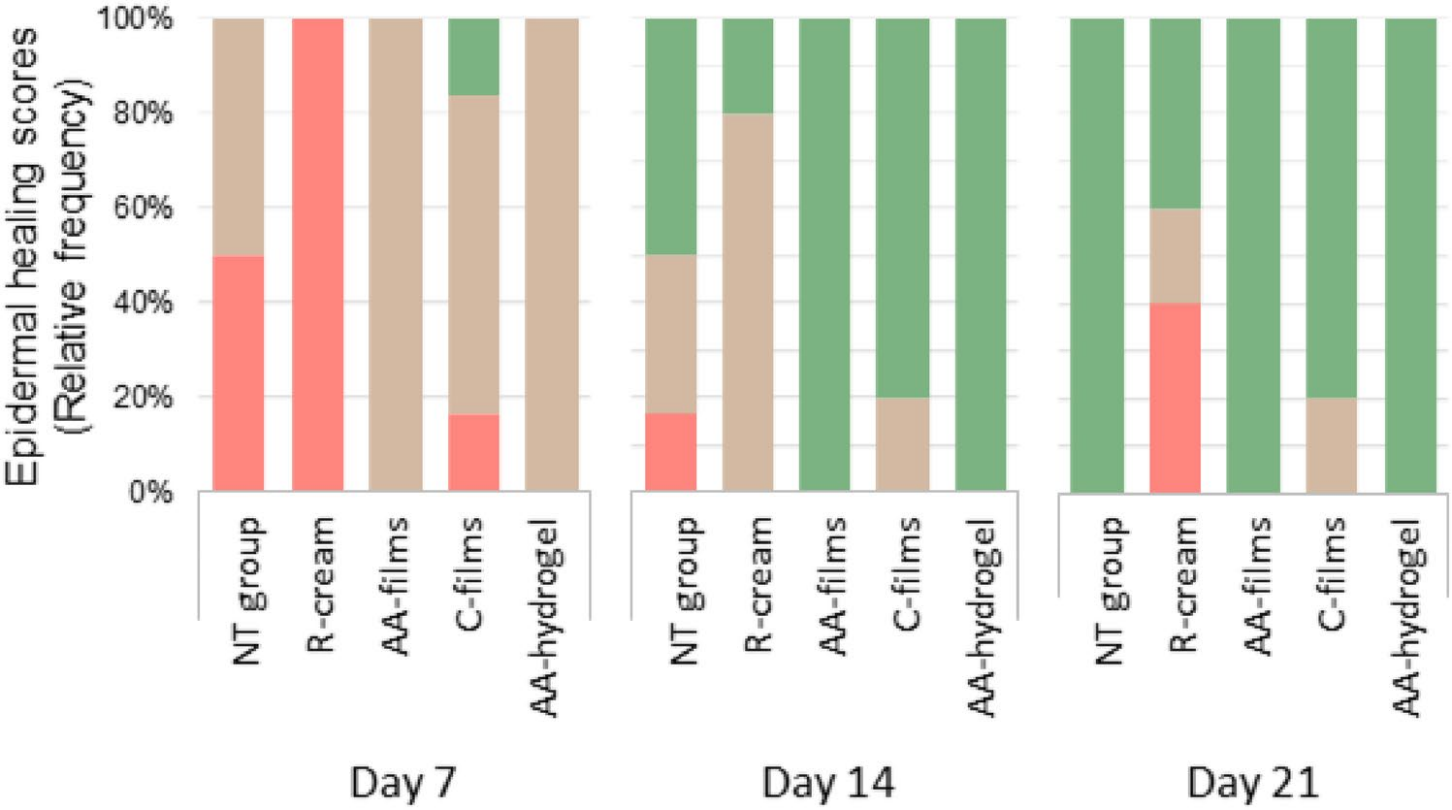
Epidermis scores were recorded as relative frequency of cases/week as: ( 1) Absent, ( 2) discontinuous and ( 3) continuous.

**Figure 7:**
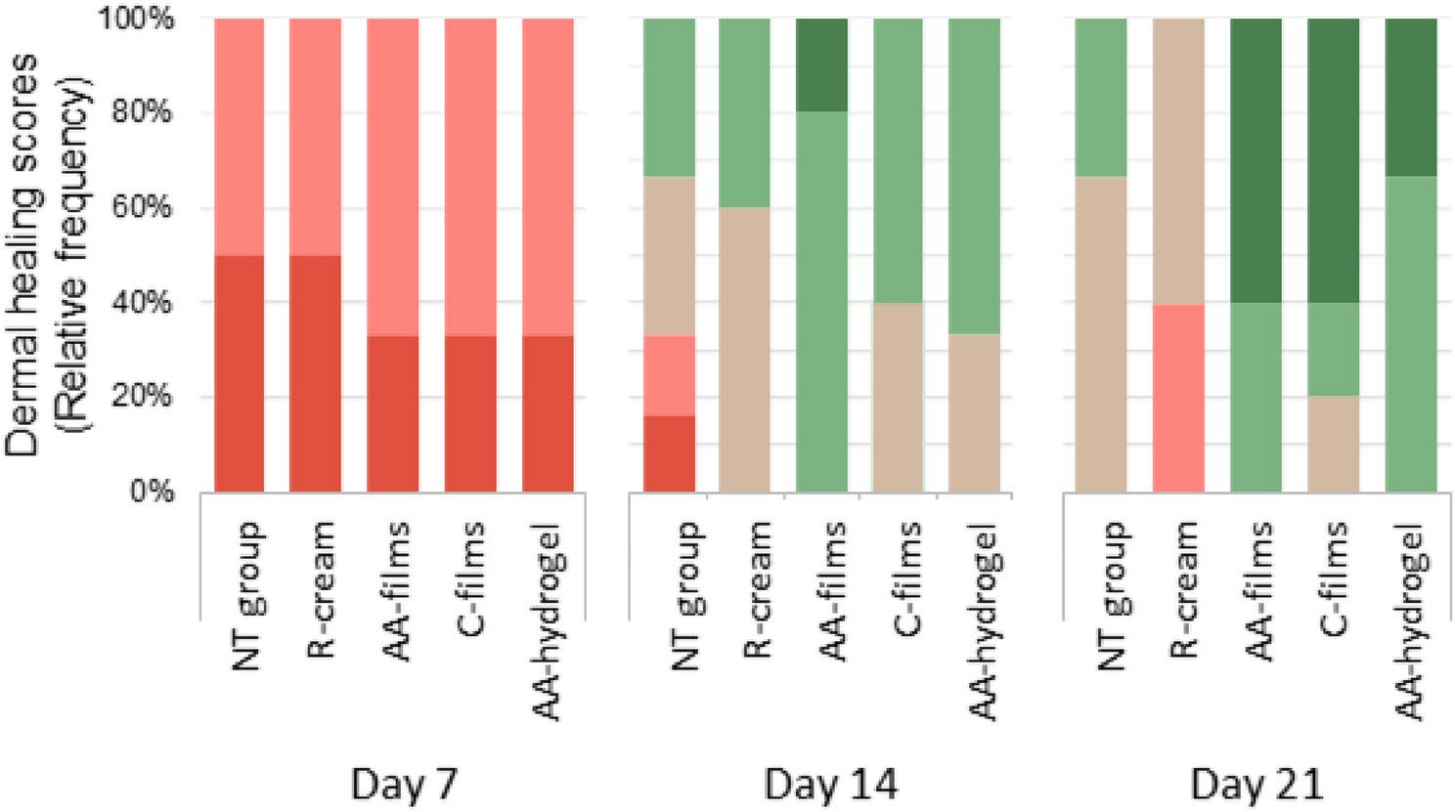
Dermis scores were recorded as relative frequency of cases/week as: ( 1) Complete dermal disorganization, ( 2) Reticular recovery, papillary disorganization, ( 3) Recovery of reticular and papillary dermis, ( 4) Normal reticular dermis with papillary recovery, ( 5) Completely normal dermis.

Figure 5 shows representative photomicrographs of each experimental group, with the epidermal evolution frequencies and dermal regeneration being shown in Figs. 6 and 7, respectively. In all cases the treatments were significantly associated with the epidermal (p < 0.005) and dermal scores (p < 0.05).

As observed in Fig. 5, on day 7 post burn, all animals treated with AA-films and AA-hydrogel presented an epidermis in regeneration that was scored as discontinuous (Fig. 6). In the same period, the epidermal recovery of the C-films group was heterogeneous, with most of them presenting discontinuous epidermis and a minority with even absent or complete epidermis. In contrast, the R-cream group revealed an absent epidermis in all cases, while the NT group showed absent or discontinuous epidermis at similar frequencies. No differences in dermal scores among AA-films, AA-hydrogel and C-films were observed, which revealed more animals with reticular recovery (score 2) than in the NT group or R-cream, with half of these groups still showing complete dermal disorganization (score 1), (Fig. 7).

On day 14, the AA-films and AA-hydrogel treatments led early to a complete epidermal healing (score 3) as well as a dermal improvement with scores 4–5 and 3–4, respectively. For both treatments, a well-differentiated epidermis having a clear basophilic staining and a corneal layer was related to the normal differentiation of reticular and papillary dermis (Fig. 5). The C-films group improved its epidermal and dermal outcomes, although to a lesser extent than the groups AA-films and AA-hydrogel. Epidermal regeneration and dermal organization in the NT group were heterogeneous, with scores ranging from 1 to 3 and 1–4, respectively. Notice that the R-cream group showed irregular epidermis and most animals showed recovery of reticular and papillary dermis (score 3) but few appendages (Figs. 6 and 7).

An interesting observation is that on day 14-post burn, the recovery of the appendages (hair follicles and associated glands) can be observed to a different extent in the dermis of AA-films, AA-hydrogel, and C-films groups. In contrast, they are absent in NT and R-cream ones. In addition, wound healing in C-films group was superior to that found in R-cream group, despite not having an antimicrobial agent in its composition. These facts demonstrated the beneficial healing effect of sodium alginate and sodium hyaluronate polysaccharides, which can be related to the moist wound environment generated by films and hydrogels that is convenient for cell growth and physiology^4,40^. Furthermore, systems based on polymers are well-known for their ability to cool the skin surface by absorbing and dissipating the heat^15,41,42^. This reduces the inflammatory response and limits tissue damage, resulting in faster regeneration^43^ and an improved well-being for the patient^43–45^. These benefits were enhanced by AA-films, which indicated that neither ciprofloxacin nor lidocaine interfered with skin healing and could actually improve it due to ciprofloxacin antimicrobial activity. This shortened evolution might prevent further development of chronic wounds and the risk of bacterial dissemination, thereby improving patient prognosis. Nevertheless, it still remains to be seen whether this system can promote faster wound healing and exhibit antimicrobial effects on an infected wound in vivo. Also, it should be taken into account that these results were obtained in a rat burn model, whose most significant limitation is the subcutaneous panniculus carnosus muscle that facilitates skin healing by both wound contraction and collagen formation. However, this rapid wound contraction allows studying the mechanics of wound healing and to compare the performance of alternative treatments^46^.

On day 21, the AA-films and AA-hydrogel reached the therapeutic goals promptly with sustained effects up to the trial end. Thus, both treatments exerted an earlier and sustained epithelialization in all cases, with the polyelectrolytes accompanied by drugs being the most effective (Figs. 5 and 6). Also, an increase in the frequency of animals with normal skin (a normal reticular dermis was found in all cases) promoted dermal remodeling respect to groups with no treatment or the standard one (Figs. 5 and 7). On the other hand, R-cream showed an inconstant and only partial epidermal recovery in the last week, even reaching lower performance than the NT group that achieved complete closure (Figs. 5 and 6). In addition, it presented skin dermal score involution (Fig. 7), despite this being considered the gold standard treatment for burns, it was significantly less effective than the other treatments. Moreover, wound healing was delayed compared with the NT group, with the skin recovery observed on day 14 being transient and with a later impairment on day 21 being accompanied by cellular infiltration (Fig. 5). This is in fact a frequent problem related to the repeated application of silver sulfadiazine creams and silver-based dressings, which are deleterious to keratinocytes and fibroblasts^7,47^, partly due to the hydrophobic nature of the vehicle and the unspecific killing action of silver^17,42,48^.

Although the epidermis was continuous in the NT group at the trial end (Fig. 7), it had an atypical structure with pale and swollen epithelia (Fig. 5), which may indicate a need for additional time to reestablish its homeostasis and cellular structure. Also, these group exhibited a low contrast between the reticular and papillary dermis as well as remaining cellular infiltration, which could evolve to a fibrous scar without skin appendages over the following days (Fig. 5).

#### Macroscopic wound healing study

**Figure 8:**
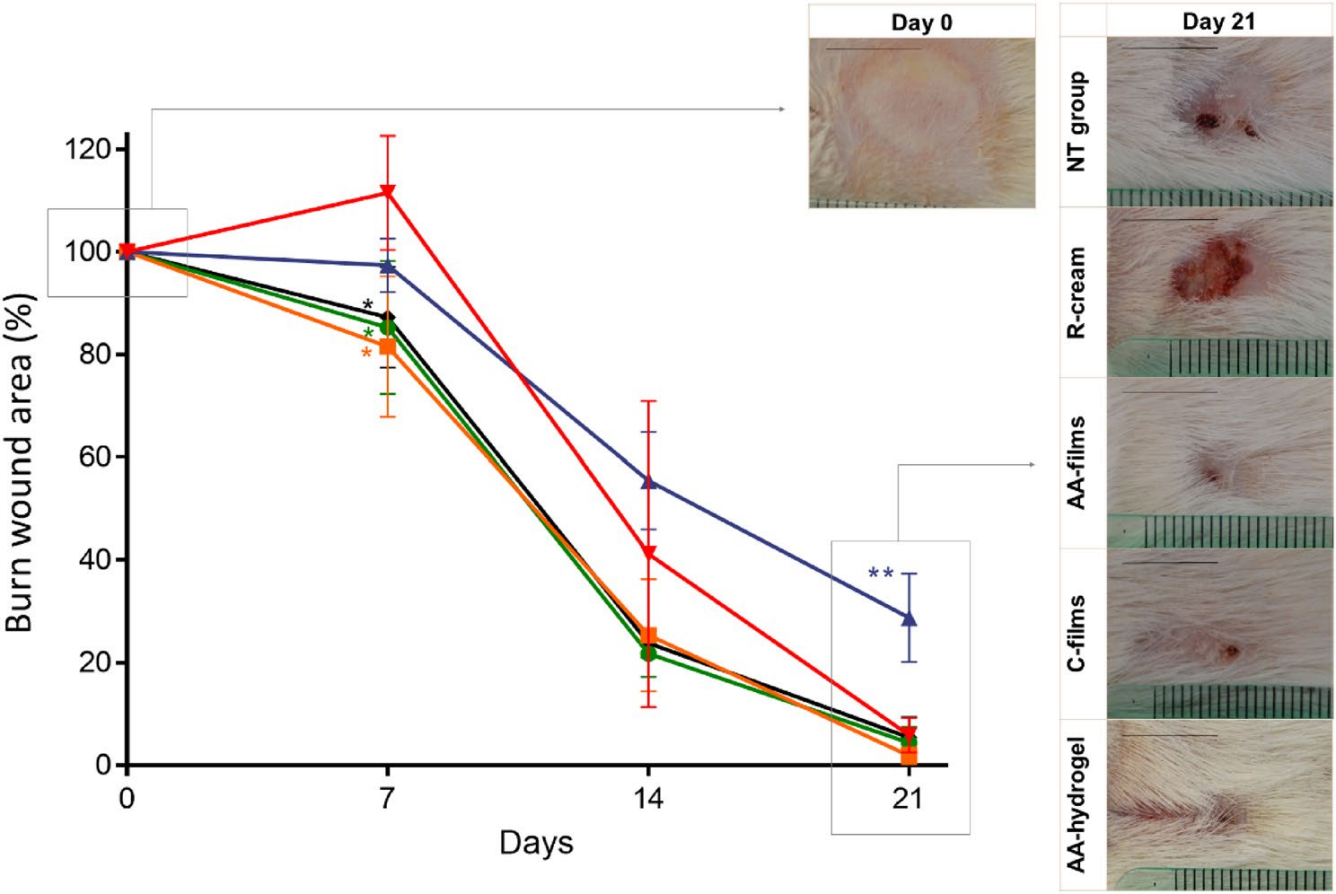
Macroscopic healing versus time: burn wound area (% with respect to initial burned area) after treatments calculated according to Eq. (3) and representative photographs. References:  AA-films,  C-films,  AA-hydrogel,  R-cream and  NT group. Scale bars are 1 cm; *significantly smaller than the NT group; **significantly larger than the NT group. Plotted with GraphPad Prism^®^ v.7.00 software (GraphPad, USA, https://www.graphpad.com/).

Although the macroscopic evaluation does not allow to see the deeper layers, it allows observing the wound closure in a global way to reflect the histological evolution. Figure 8 and Supplementary Fig. S2 shows the wound healing results for each treatment after 0, 7, 14, and 21 days-post burn.

Figure 8 revealed that only the AA-films, AA-hydrogel, and C-films groups showed a significant reduction in wound areas on day 7 respect to NT group (p < 0.05), with the R-cream group showing no differences in wound area, whereas it slightly increased in the NT group, possibly due to local dermal infiltration observed in the corresponding biopsy (Fig. 5). On day 14, a further wound area reduction was observed in the AA-films, AA-hydrogel and C-films groups. R-cream was less effective in closing wounds than the other treatments or even than NT group (p < 0.05). However, relevant differences that are not seen macroscopically can be seen histologically, as already discussed. On day 21, all groups except for R-cream presented almost complete wound closure with no macroscopic differences been observable with respect to the NT group. However, the R-cream group revealed a higher wound area at the trial end, even with respect to the NT group (p < 0.05). These results, although less precise than histologic observations, confirm the faster reepithelization induced by the film/hydrogel treatments. Besides, the harmful effects of R-cream were also confirmed.

It should be noted that initial lesions (Fig. 8, day 0) showed a clear burned area without hair. However, at the end of the treatments (day 21th) the AA-films, C-films and AA-hydrogel groups showed a large amount of hair which is an indicator of skin healing. By the contrary, a lower density of hairs is observed in the NT group, which is even lower in the R-cream group. Figure S2 shows an enlarged and more complete set of representative photographs of macroscopic healing, including AA-films and C-films groups on day 21st with trimmed hair, which allowed to accurately assess wound healing without being compromised by the presence of hair over the regrown skin. Figure S2A shows that the center of the wound is completely closed in AA-films group while an extensive but not complete wound closure is appreciated in representative C-films group, with no inflammatory signs or other relevant findings. These observations are in agreement with both the better microscopic epidermal healing and dermal healing scores obtained for AA-films group as compared with C-films group (Figs. 6 and 7) at day 21. The presence/absence of hair is also consistent with the histological results shown in Fig. 5, where hair follicles and other appendage increased in the healed area under a complete epithelial recovery but are absent in NT- and R-cream groups. In line with that, Oryan et al.^12^ observed the absence of hair follicles and other appendages in wounds treated with 1% sulfadiazine cream but hair growing in those treated with chitosan-capped silver nanoparticles, which was assigned to an increased compatibility with the tissue allowing wound regeneration.

The present investigation took advantage of the ionic interaction established between a polyelectrolyte and certain drugs to develop new materials with potential applications in clinic. Although both the AA-films and AA-hydrogel promoted skin healing, the films show more advantages as wound dressings, such as their easy and non-invasive application and dose accuracy. The development or optimization of advanced dressings still represents a very active research field, with the aim being to improve skin healing in relation to specific clinical applications. Related to this, numerous new systems containing peptides, growth factors or cells^37,49,50^ have been proposed in the scientific literature as improved alternatives for the treatment of wounds in general, and have demonstrated the ability to increase wound healing. Compared with these antecedents, the AA-films development has revealed several advantages, such as being an economic, simple and robust manufacturing process accomplished by the rational selection of materials and the knowledge of their chemical and biological characteristics. It can thus be suggested that the design films made from biomaterials with antimicrobials and anesthetics with extended-release and a sound manufacturing process is a promising development for the improvement of burn pharmacotherapy. Moreover, from a technological and pharmaceutical point of view, the use of these biomaterials and drugs approved by regulatory health authorities constitutes a translational medicine approach with a great potential for advancing to the productive sector, with the possibility of becoming a new alternative use of the drugs.

## Materials and methods

### Materials

Ciprofloxacin hydrochloride was donated by Bago laboratory (Argentina). Cip as a base was obtained from ciprofloxacin hydrochloride according to Sanchez et al.^18^. Lidocaine hydrochloride, Carbomer 974P NF (CB), CaCl_2_ and sodium hyaluronate (SH) were obtained from Parafarm (Argentina). Sodium alginate (SA) was purchased from Sigma Aldrich (USA). Anhydrous glycerol 99.5% was obtained from Cicarelli (Argentina). The 0.9% NaCl sterile solution was from Braun (Argentina), and ethanol 70 v/v was diluted from 96 v/v (Bialcohol, Porta, Argentina) according the Argentinian Pharmacopeia, 7th edition (FA7)^51^. All these chemical reagents and solvents used were of pharmaceutical or analytical grade. Ultrapure water was obtained from a water purification system (Heal Force, China) and sterilized.

### Preparation of PE-Cip systems for cell culture

The PE-Cip systems were obtained as follows: water dispersions of PE (CB, SA or SH) were prepared at different concentrations. Then, an aqueous suspension of Cip was added under stirring until the disappearance of the Cip particles. Also, dispersions of PE alone or of CipHCl solutions (in equivalent Cip concentrations) were obtained. Finally, 1:10 dilutions were made with culture medium (Table 1).

**Table 1:** PE-Cip treatments.

Assay	Cip concentration	PE*	PE_L_-Cip systems	PE_H_-Cip systems
Cell viability	3 µg/ml (Cip_3_)	0.01% (PE_L_)	PE_L_-Cip_3_	PE_H_-Cip_3_
	30 µg/ml (Cip_30_)	0.1% (PE_H_)	PE_L_-Cip_30_	PE_H_-Cip_30_
Wound healing scratch	80 µg/ml (Cip_80_)**	0.1% (PE_H_)	–	PE_H_-Cip_80_

*PE are CB, SA, or SH; PE_L_ (means PE low concentration) and PE_H_ (means PE high concentration).

**Due to the greater number of cells per plate required for the wound healing scratch assay, the highest concentration of Cip that was able to dissolve in the culture medium was used^52^.

### Cell culture

Normal Human Dermal Fibroblasts (HDF) were obtained from anonymous healthy volunteers (age, who informed consent to the use of leftover tissue from cosmetic surgeries, with the protocol being approved by the Research Ethics Committee of the Hospital Nacional de Clínicas, Universidad Nacional de Córdoba (registration code: 728-2018) in compliance with the Córdoba’s Law 9694^53^.

The cultivated cells were used between passages 4 and 6. HDF cells were cultivated in Dulbecco’s Modified Eagle’s Medium (DMEM, Gibco, USA) supplemented with 20% (v/v) fetal bovine serum (FBS, Natocor, Argentina), 100 µg/ml penicillin and 100 µg/ml streptomycin (Invitrogen, USA) at 37 °C and 5% CO_2_ (SANYO MCO-17AC, Germany).

### Study of cell compatibility: cell viability assay

To evaluate cell viability, 1 × 10^6^ cells/plate were cultured in 96-well plates (∼ 80% of confluence) for 24 h. Then, the cells were treated with different PE-Cip conditions (Table 1) for 24 h, and cell viability was evaluated using the metabolic dye 3-(4,5-dimethylthiazol-2-yl)-2,5-diphenyl tetrazolium bromide (MTT) assay. Briefly, an MTT solution (1 mg/ml MTT in phosphate buffered saline, Sigma-Aldrich, USA) was added to each well (1:10) and incubated for 2.5 h at 37 °C. After this incubation, the media were removed and the formazan precipitated was dissolved in 100 μl of dimethylsulfoxide (DMSO, Cicarelli, Argentina). Absorbance was measured at 540 nm with a photometer (Glomax, Promega, USA), and the results were expressed as the percentage of cell viability (CV%) relative to the control, according Eq. (1). Two independent experiments were conducted in quadruplicate (n = 8).

1 $$CV\mathrm{\% }= \frac{\left[{A}_{sample}\right]}{\left[{A}_{control}\right]}\times 100\mathrm{\%}$$

where [A_sample_] and [A_control_] represent the absorbance measured in treated and untreated cells, respectively.

According to ISO 10993-5^54^, “Biological evaluation of medical devices. Part 5: Tests for in vitro cytotoxicity”, the lower the CV % value, the higher the cytotoxic potential of the test item is. If viability is reduced to < 70% of the blank, it has a cytotoxic potential.

The statistical analysis was carried out using GraphPad Prism v.7.00 software (GraphPad, USA, https://www.graphpad.com/). For the cell viability assay, data were expressed as the mean ± SEM (n = 8). The differences among the treatments were analyzed by ANOVA and Tukey’s post hoc test.

### Effect of PE and its PE-Cip complexes on HDF cell migration

#### Wound healing scratch assay in cell culture

The migratory potential of fibroblasts was assessed by a wound healing scratch assay as described by Flores-Martín et al.^55^. Briefly, 3 × 10^5^ cells/plate were cultured in 24-well plates for 24 h, after which, two parallel scratches were made in the confluent monolayer of each well with a plastic disposable pipette tip (10 µL). The cultures were washed twice with phosphate buffered saline (PBS, Sigma, USA) to remove all detached cells, and cultivated under different PE-Cip conditions (the culture medium had only 2% of SFB in order to minimize cell growth) for 24 h (Table 1).

The wound area was photographed at t = 0 h and t = 24 h, with a microscope coupled to a DMI 8 digital camera (LEICA, Germany). The pictures were analyzed using ImageJ v.1.51c software (NIH, USA, https://imagej.nih.gov/ij/), and the wound healing was expressed as migrated area % (MA%) and was calculated according Eq. (2). Three independent experiments were conducted in quadruplicate (n = 12).

2 $$MA \mathrm{\% }= \frac{\left[{Area}_{24 h}\right]}{\left[{Area}_{0 h}\right]}\times 100\mathrm{\%}$$

where [Area_0 h_] is the area at t = 0 and [Area_24 h_] is the cell-populated area after 24 h of the test. MA% vs treatments were plotted with GraphPad Prism v.7.00 software (GraphPad, USA, https://www.graphpad.com/).

#### Effect of PE and its PE-Cip complexes on the protein expression implicated in cell migration. SDS-PAGE and Western Blot

Whole protein extracts were prepared in sample buffer (2 × Laemmli buffer containing 2-β-mercaptoethanol, Bio-Rad, USA) as described in Flores-Martín et al.^55^, and protein samples were loaded onto a 10% SDS-PAGE gel. After migration, the proteins were electrotransferred to nitrocellulose membranes (Amersham Bioscience, USA). A molecular weight marker (MWM, sds7b2 Sigma-Aldrich, USA) was included to identify the approximate size of the proteins on the gels. The full length membranes were stained with Ponceau S Staining Solution (Thermo Fisher, USA) and were cut before hybridization with the specific antibodies. The membranes were blocked for 1 h with 5% v/v non-fat dry milk in Tris buffered saline (TBST) (25 mM Tris, 150 mM NaCl, 2 mM KCl, pH 7.4, containing 0.2% v/v Tween-20), washed, and incubated with each of the following primary antibodies: mouse monoclonal anti-integrin β1 (dilution 1/300 v/v; sc-374429 Santa Cruz Biotechnology, USA); rabbit polyclonal anti-p-Fak Tyr 397 (dilution 1/200 v/v; sc-11765-R, Santa Cruz Biotechnology, USA); and mouse monoclonal anti-α-tubulin (dilution 1/5000 v/v; clon B-5-1-2, Sigma-Aldrich, USA). After washing with TBST, blots were incubated with IRDye 800CW donkey anti-rabbit IgG (1:10000; P/N 926-32213, LI-COR Biosciences, USA) or IRDye 680RD donkey anti-mouse IgG (1:10000; P/N 926-68072, LI-COR Biosciences, USA) antibodies in TBS for 1 h, protected from light. After two washes with TBST and one with TBS, the membranes were visualized and quantified using the Odyssey Infrared Imaging System (LI-COR, Inc., USA). Protein expression was normalized to the α-tubulin levels. The relative levels of proteins vs treatments were plotted with GraphPad Prism v.7.00 software (GraphPad, USA, https://www.graphpad.com/).

##### Statistical analysis

In the wound healing scratch assay, the data were expressed as the mean ± SEM (n = 12). The differences among the treatments were analyzed by Kruskal–Wallis and Dunn’s post hoc test. In SDS-PAGE and *Western Blot*, the data were expressed as the mean ± SEM (n = 6). The differences among these treatments were analyzed using ANOVA and Dunnett’s post hoc test utilizing GraphPad Prism v.7.00 software (GraphPad, USA, https://www.graphpad.com/). In all cases, p < 0.05 was considered statistically significant.

### Preparation of the AA-films and AA-hydrogel for the in vivo assay

The AA-films were prepared by the casting technique according to Sanchez et al.^20^. For comparison, the same hydrogel, but without the drugs, was also prepared and used as a control (C-films)^20^. The AA-hydrogel was prepared according to Sanchez et al.^18^. A cream with 1.00% silver sulfadiazine, 0.66% lidocaine and 248000 UI vitamin A (Platsul A cream, Souberian Chobet S.R.L., Batch nº 11318, Argentina) was used as a reference treatment (R-cream).

### Wound-healing in vivo test

An experimental murine model of deep second degree burns was used^56^. The studies were conducted according to ethical protocols described in the National Institutes Health guide for the care and use of experimental animals^57^ and in compliance with the ARRIVE guidelines. In addition, the trial was approved by the Ethics Committee CICUAL of the University of La Rioja (Protocol nº 5/18).

Healthy male Wistar rats, with a weight of between 250 and 300 g, were placed into cages with a physical division to avoid contact between animals, kept in a room with controlled temperature (21 ± 5 °C), and exposed to light/dark cycles of 12 h. To minimize the risk of infection, the wood sawdust of the cages was changed three times per week. Animals had free access to food and water.

On day 0, the rats were anesthetized with an intraperitoneal injection of 85.0 mg/kg ketamine (Ketamina 50, 50 mg/ml, Holliday-Scott, Argentina) plus 6.0 mg/kg xylazine (Xilacina 20, 20 mg/ml Richmond, Argentina), according to Yaman et al.^56^. Then, the lower dorsal region of each animal was shaved and then cleaned with 70 v/v ethanol. A deep second-degree burn was made by exposure to a metal device for 30 s without additional pressure (cylinder 1 cm in diameter, 10 g, pre-heated by immersion in boiling water). The metal device temperature (90 ± 2) °C was measured with the infrared thermometer ST882 (Reed Instruments, USA). Finally, the burns were cleaned with sterile gauze soaked in 0.9% NaCl sterile solution. Immediately after, the animals were randomly divided into the 5 groups as described below in Table 2.

**Table 2:** Assignment of experimental treatments.

Group	Nº animals
AA-films	6
C-films (control, drug-free films)	6
AA-hydrogel	3
R-cream (reference treatment)	6
NT group (no treatment, negative control)	6

Animals of the AA-film and C-film groups were treated with a circular portion of these films cut with a 12 mm ø punch. The AA-hydrogel and R-cream groups were treated with 0.2 ml of the corresponding treatment applied with a 1-ml syringe. Before each treatment application, the wounds were cleaned with sterile gauze soaked in 0.9% NaCl solution. The treatments were repeated once a day for 21 days. Investigators could not be blinded because the topical treatments were easily distinguishable from each other; i.e. R-cream is a white semisolid, AA-hydrogel is a transparent semisolid, and AA-films and C-films are solid transparent circular portions of films. At the end of the experiment, the animals were sacrificed with a lethal dose of the anesthetics.

#### Microscopic wound healing in vivo study

**Figure 9:**
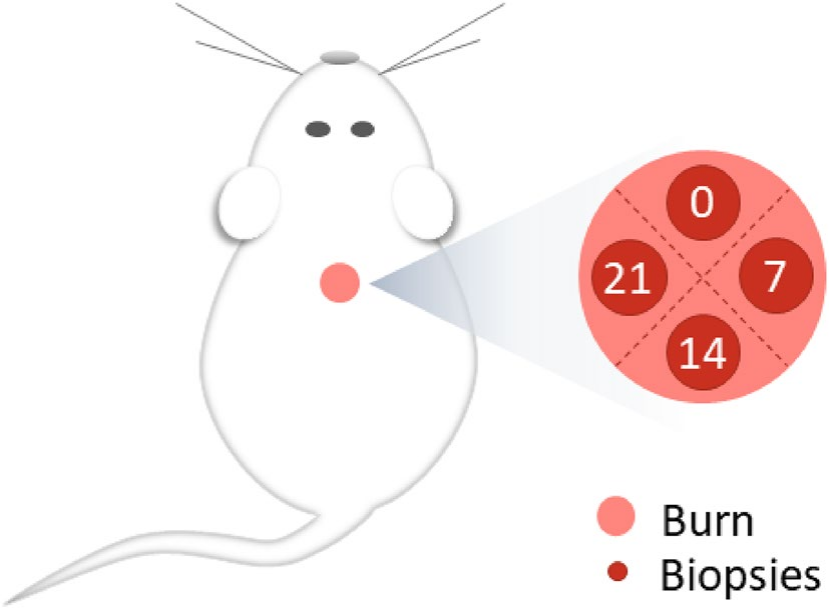
Biopsy location. The wounds were divided into four parts with two imaginary lines. Biopsies were taken clockwise from the center of each part (according to day 0) on days 0, 7, 14, and 21. Thus, each animal was its own control, and in this way, we managed to reduce the number of experimental animals used, according to the Guide for the Care and Use of Experimental Animals.

Burned skin tissue biopsies (3 mm) were collected under anesthesia on days 0, 7, 14, or 21, as shown in Fig. 9. These samples were fixed in 10% neutral buffered formalin (Biopack, Argentina), dehydrated by serial immersion, embedded in paraffin, and cut with a microtome into 5 μm sections. These sections were subsequently processed using the hematoxylin–eosin universal technique. Then, photomicrographs were taken at 100× magnification using the BX41 optical light microscope (Olympus, Japan).

The epidermal continuity and dermal regeneration were obtained from 6 fields of each biopsy and were scored as the relative frequency of cases according to Table 3.

**Table 3:** Epidermal and dermal histological assessment scores.

Tissue	Score	Description
Epidermal continuity	1	Absent
	2	Discontinuous
	3	Continuous
Dermal recuperation	1	Complete dermal disorganization
	2	Reticular recovery, papillary disorganization
	3	Recovery of reticular and papillary dermis
	4	Normal reticular dermis with papillary recovery
	5	Completely normal dermis

The Chi-square method was used to establish statistical associations between the histological scores and treatments. The Cochran-Mantel–Haenszel test was used in the time-stratified analysis of the epidermal and dermal scores to evaluate the association between treatments and score frequencies for p < 0.05. Statistical probes were performed using the InfoStat v.2012 software (InfoStat Group, Argentina, https://www.infostat.com.ar/).

#### Macroscopic wound healing in vivo study

Animal behavior was observed each day. Also, the appearance, color, hair growth and clinical signs of a possible infection process (heat, redness, and swelling) of the wounds were recorded. On days 0, 7, 14, and 21, the wounds were photographed with a d3200 camera (Nikon, Japan), with a ruler being placed for enable further analysis. The burn wound area was measured from these photographs using Image J v1.51j8 software (NIH, USA, https://imagej.nih.gov/ij/), and the wound healing was calculated according to Eq. (3):

3 $$Macroscopic\;wound\;healing\;(\%) = \frac{\left[{Area}_{t}\right]}{\left[{Area}_{ 0}\right]}\times 100\%$$

where *[Area*_*0*_*]* is the wound area on day 0 and *[Area*_*t*_*]* is the wound area on days 7, 14 or 21. The mean macroscopic wound healing ± SD (n = 6) versus time was plotted. Treatments were compared at each time recorded using ANOVA followed by Fisher’s test with a significance level of p < 0.05, utilizing InfoStat v.2012 software (InfoStat Group, Argentina, https://www.infostat.com.ar/).

## Supplementary Information

Supplementary Figures.

## Data Availability

The datasets used and/or analysed during the current study available from the corresponding author on reasonable request.
