# Supplementary material for: Ionic complexation improves wound healing in deep second-degree burns and reduces in-vitro ciprofloxacin cytotoxicity in fibroblasts
Source: Sci Rep. 2022 Sep 26;12:16035. doi: 10.1038/s41598-022-19969-w (PMC9513095; doi:10.1038/s41598-022-19969-w)
Supplement: Supplementary file 1 — Supplementary Figures. [file 41598_2022_19969_MOESM1_ESM.docx]

Supplementary Material

**Ionic complexation improves wound healing in deep second-degree burns and reduces in‑vitro ciprofloxacin cytotoxicity in fibroblasts**

Sanchez, María Florencia; Guzman, María Laura; Flores-Martín, Jesica; Cruz Del Puerto, Mariano; Laino, Carlos; Soria, Elio Andrés; Donadio, Ana Carolina, Genti-Raimondi, Susana; Olivera, María Eugenia.


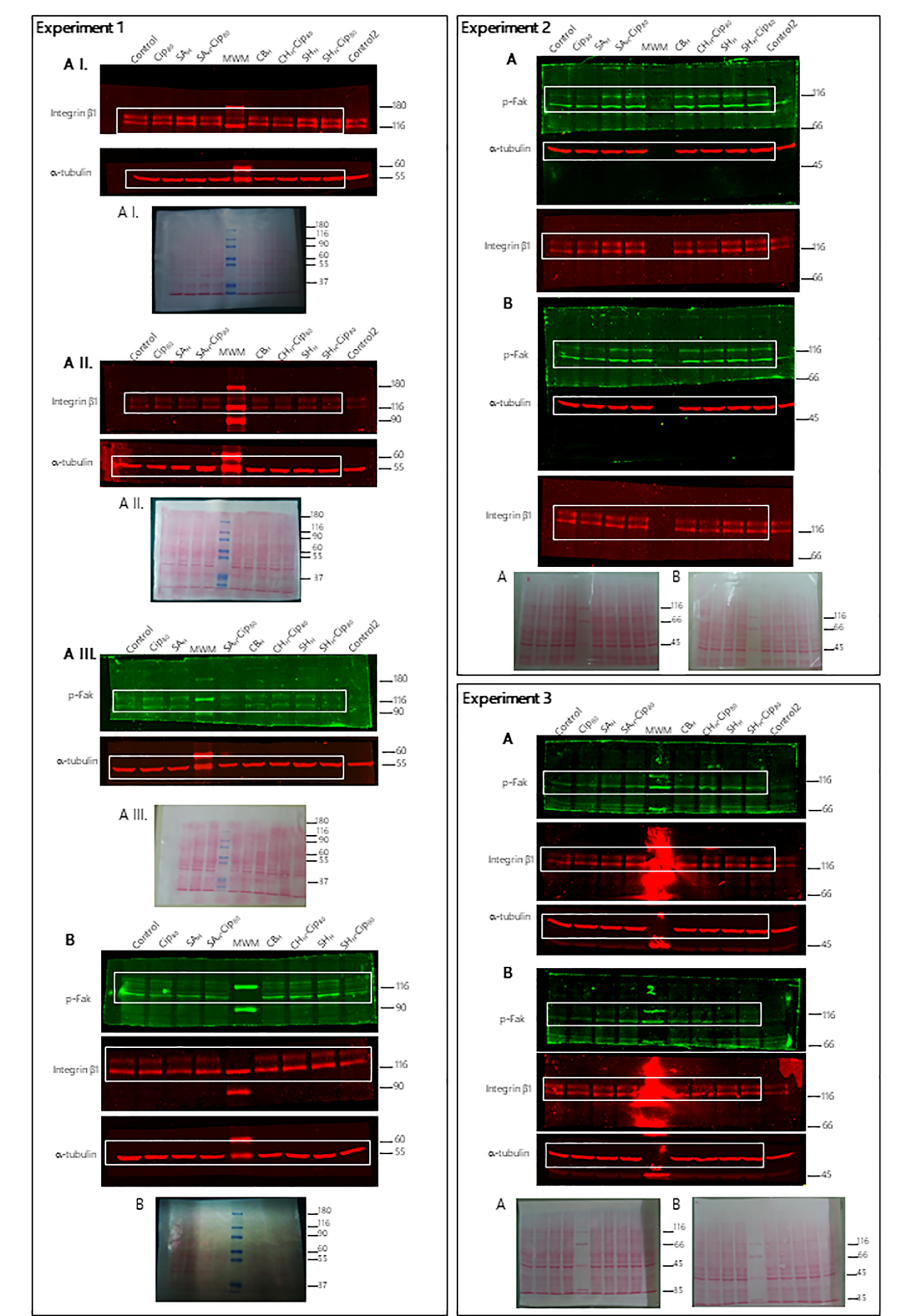


**Supplementary Fig. S1.** Western blots performed from protein extracts of three independent experiments by duplicates (A and B).  The membranes were cut before hybridization with the specific antibodies. The full length membranes staining with Ponceau S are shown below each experiment and the molecular weight markers used are indicated on the right. The western blot from experiment 1 A was repeated 3 times (I, II, and III). The white boxes show the bands that were quantified. Control 2 was not taken into account. The membranes from experiment 1AI and 2A were chosen to show in Figure 4A. MWM (molecular weight marker).

**
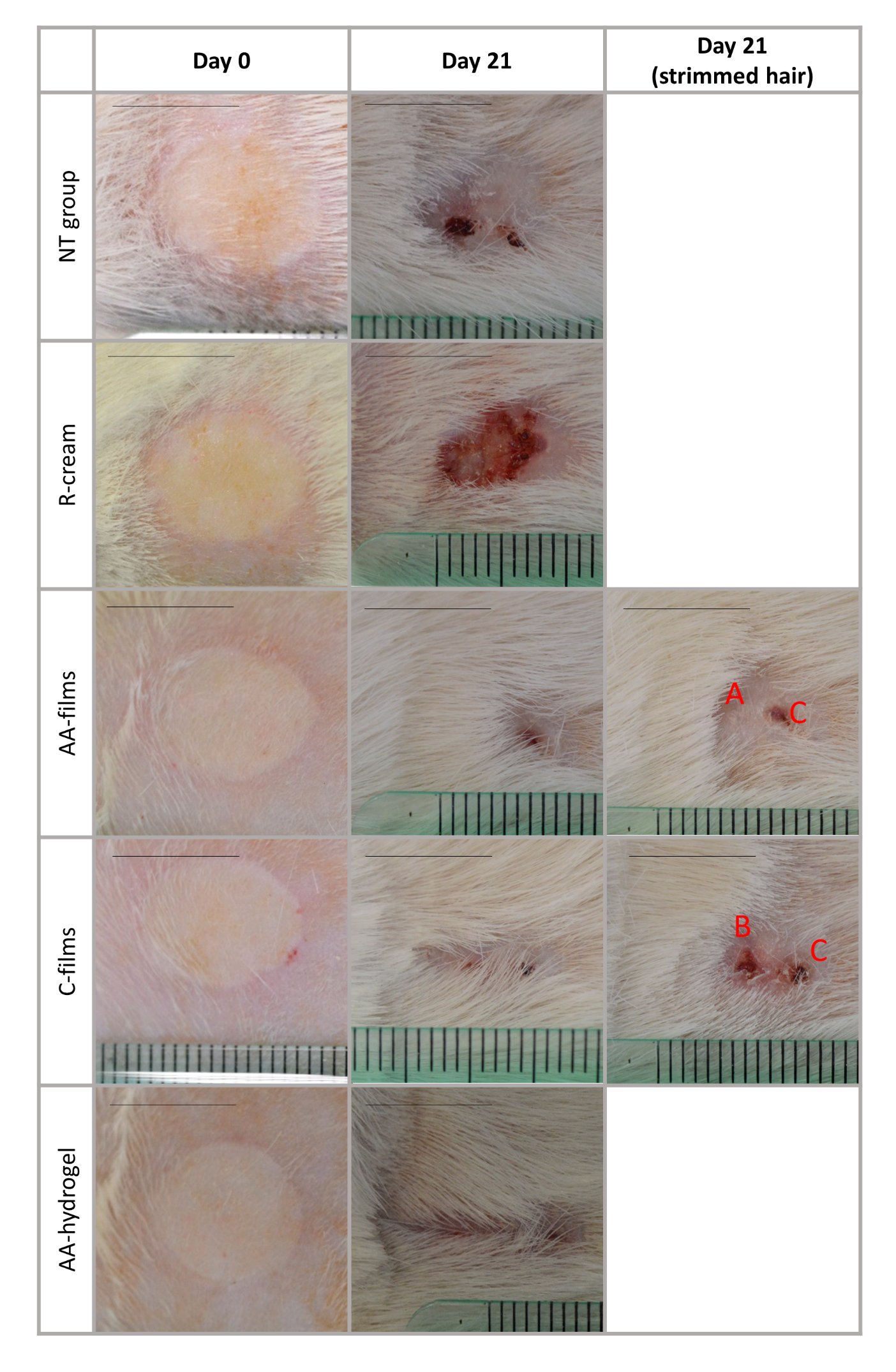
**

**Supplementary Fig. S2.** Representative photographs of macroscopic healing immediately after burning and at the end of the trial. For a better visualization of the healed skin on day 21, the hair was trimmed in AA-film and C-film groups. (A) The center of the wound is completely closed in AA-film group, (B) the central part of the wound is not completely closed yet in C-film group, (C) lesions corresponding to the biopsy taken on day 14 (see methodology section). Scale bars are 1 cm.
